# Supplementary material for: Monocarboxylate Transporter 1 (MCT1) in Liver Pathology
Source: Int J Mol Sci. 2020 Feb 26;21(5):1606. doi: 10.3390/ijms21051606 (PMC7084425; doi:10.3390/ijms21051606)
Supplement: Supplementary file 1 [file ijms-21-01606-s001.pdf]

Supplementary material

Monocarboxylate transporter 1 (MCT1) in liver pathology

Marek Droździk, Sylwia Szeląg-Pieniek, Justyna Grzegółkowska, Joanna Łapczuk-Romańska, Mariola Post, Pawel Domagała, Janusz Miętkiewski, Stefan Oswald, Mateusz Kurzawski

Supplementary table 1. Expression of *SLC16A1* (relative mRNA quantity) in the liver samples.

	**mean**	**SD**	**CV%**	**median**	**min**	**max**
**CTRL** (n=20)	1.00	0.47	47	1.04	0.24	2.35
**Liver pathology** (n=77)*	0.71	0.45	64	0.54	0.16	2.15
**HCV** (n=21)	0.88	0.54	61	0.74	0.24	2.11
**PBC** (n=10)*	0.44	0.18	40	0.41	0.18	0.82
**PSC** (n=6)	0.62	0.23	36	0.55	0.37	1.12
**ALD** (n=20)*	0.54	0.29	54	0.44	0.20	1.32
**AIH** (n=20)	0.80	0.48	60	0.62	0.16	1.83

control livers (CTRL), hepatitis C (HCV), primary biliary cholangitis (PBC), primary sclerosing cholangitis (PSC), alcoholic liver disease (ALD) and autoimmune hepatitis (AIH); SD - standard deviation; CV%- coefficient of variation; min-minimum; max-maximum; * significant difference in relation to control group $p<0.05$;

Supplementary table 2. Expression of *SLC16A1* (relative mRNA quantity) in different stages of liver disease.

	**mean**	**SD**	**CV%**	**median**	**min**	**max**
**CTRL**	1.00	0.47	47	1.04	0.24	2.35
**A**	0.79	0.42	53	0.65	0.16	1.74
**B**	0.75	0.42	56	0.71	0.16	2.00
**C***	0.61	0.48	79	0.44	0.20	2.15

SD- standard deviation; CV%- coefficient of variation; min-minimum; max-maximum; CTRL – control livers; A, B, C - Child-Pugh stages of liver disease; * significant difference in relation to control group $p<0.05$;

Supplementary table 3. MCT1 protein quantity (fmol/mg tissue) in liver samples.

	mean	SD	CV%	median	min	max	positive
**CTRL** (n=20)	115.7	85.3	74	103.7	17.9	346.1	20/20
**Liver pathology** (n=77)*	58.4	46.6	79	56.8	0.0	306.1	75/77
**HCV** (n=21)	52.1	41.6	80	40.6	0.0	167.0	20/21
**PBC** (n=10)	63.6	24.7	39	58.0	28.7	112.5	10/10
**PSC** (n=6)	60.3	12.0	20	64.2	37.1	78.5	7/7
**ALD** (n=20)*	45.8	45.4	99	26.1	1.7	167.6	20/20
**AIH** (n=20)	74.1	59.8	81	62.9	0.0	306.1	19/20

control livers (CTRL), hepatitis C (HCV), primary biliary cholangitis (PBC), primary sclerosing cholangitis (PSC), alcoholic liver disease (ALD) and autoimmune hepatitis (AIH); *significant difference in relation to control group p<0.05;

Supplementary table 4. MCT1 protein quantity (fmol/mg tissue) in different stages of liver disease.

	**mean**	**SD**	**CV%**	**median**	**min**	**max**	**positive samples**
**CTRL**	115.7	85.3	74	103.8	17.9	346.1	20/20
**A**	56.7	28.4	50	54.8	7.1	134.8	18/18
**B**	66.4	61.1	92	63.8	0.0	306.1	28/30
**C***	50.7	33.4	66	44.5	7.6	167.6	28/28

SD- standard deviation; CV%- coefficient of variation; min-minimum; max-maximum; positive samples- number of samples with detectable level of the protein (>0.1 fmol/mg); CTRL – control livers; A, B ,C - Child-Pugh stages of liver disease; *significant difference in relation to control group p<0.05;

Supplementary table 5. Correlation analysis (Spearman coefficient, r) between *SLC16A1* mRNA and MCT1 protein quantity in the liver pathologies and in different stages of liver disease.

**mRNA vs. protein**	**r**	***p***
**CTRL**	-0.072	0.762
**Liver pathology**	0.083	0.420
**ALD**	0.017	0.945
**AIH**	0.136	0.567
**HCV**	-0.075	0.746
**PBC**	0.370	0.293
**PSC**	-0.257	0.623
**A**	-0.263	0.291
**B**	-0.076	0.686
**C**	0.255	0.190

control livers (CTRL), hepatitis C (HCV), primary biliary cholangitis (PBC), primary sclerosing cholangitis (PSC), alcoholic liver disease (ALD) and autoimmune hepatitis (AIH); A, B ,C - Child-Pugh stages of liver disease
